# Small Animals, Big Impact? Early Farmers and Pre- and Post-Harvest Pests from the Middle Neolithic Site of Les Bagnoles in the South-East of France (L’Isle-sur-la-Sorgue, Vaucluse, Provence-Alpes-Côte-d’Azur)

**DOI:** 10.3390/ani12121511

**Published:** 2022-06-09

**Authors:** Simone Häberle, Marguerita Schäfer, Raül Soteras, Héctor Martínez-Grau, Irka Hajdas, Stefanie Jacomet, Brigitte Röder, Jörg Schibler, Samuel van Willigen, Ferran Antolín

**Affiliations:** 1 Department of Environmental Sciences, Integrative Prehistory and Archaeological Science (IPAS), Basel University, 4055 Basel, Switzerland; raul.soteras@dainst.de (R.S.); hector.martinezgrau@unibas.ch (H.M.-G.); stefanie.jacomet@unibas.ch (S.J.); joerg.schibler@unibas.ch (J.S.); ferran.antolin@dainst.de (F.A.); 2 Deutsches Archäologisches Institut, Referat Naturwissenschaften an der Zentrale, 14195 Berlin, Germany; 3 Laboratory of Ion Beam Physics (LIP), Swiss Federal Institute of Technology (ETH) Zurich, 8093 Zurich, Switzerland; hajdas@phys.ethz.ch; 4 Departement Altertumswissenschaften, Ur- und Frühgeschichtliche und Provinzialrömische Archäologie, Basel University, 4051 Basel, Switzerland; brigitte.roeder@unibas.ch; 5 InSitu Archéologie, 1950 Sion, Switzerland; samuel.vanwilligen@insitu-archeo.ch

**Keywords:** microfauna, wood mice, grain weevil, pests, resource stress, risk management, Middle Neolithic, western Mediterranean

## Abstract

**Simple Summary:**

Archaeological excavations at Les Bagnoles (SE France) unearthed three Middle Neolithic water wells (4250–3700 cal B.C.) whose waterlogged conditions allowed the preservation of organic remains, notably a large number of rodent and beetle species. The evaluation of these results in the context of an open-air farming site raised the question of whether these species could have posed a threat to both the field plants and crops stored at the Middle Neolithic settlement. The findings of this study suggest that the native wood mouse was probably one of the first unwanted inhabitants of human settlements in Europe prior to the appearance of the invasive house mouse during the Bronze Age. The analysis also advances the argument that the storage pest grain weevil played a role in the clear shift in the crop spectrum from naked to glume wheat around 4000 B.C., as glume wheats are less vulnerable to its infestation. Moreover, the current study demonstrates that investigating insects and rodents from archaeological sites is key to grasping the challenges of the competitive relationship between Neolithic farming communities and pests.

**Abstract:**

Pests appear to have accompanied humans and their crops since the beginning of farming. Nevertheless, their study is only rarely integrated into research on farming in prehistory. An assemblage of invertebrates and small mammals was recovered from the waterlogged layers of three wells at the Middle Neolithic site (4250–3700 cal B.C.) of Les Bagnoles (SE France). The microfaunal remains were retrieved from sediment samples by wet sieving (wash-over technique). The most common among the rodents is the wood mouse. The assemblage also consists of insect remains of grain weevil, seed beetle, and corn ground beetle. The different finds not only offer data on the role of insect and rodent pests in the Neolithic but on the possible strategies adopted by the early farming communities in the western Mediterranean in response to pest infestation. The findings appear to confirm the hypothesis that the wood mouse was a commensal and storage pest in settlements long before the arrival of the invasive house mouse during the Bronze Age. The presence of the main storage pest, the grain weevil, suggests a long-term grain storage issue at Les Bagnoles. The combination of the results of the site’s archaeobotanical findings with those of other sites in the western Mediterranean suggests that the shift from naked to glume wheat around 4000 B.C. may also stem from a reaction to the problem of grain weevil infestation.

## 1. Introduction

The sedentary way of life of the Neolithic linked to animal husbandry and farming provided a more predictable source of food than in previous periods. Nevertheless, to ensure their survival, early farming communities had to counter challenging factors such as climatic and weather fluctuations, periods of drought or flooding, variations of annual temperatures [1,2], different types of soil from region to region, and altered seasonal access to water [3] by resorting to different resilience strategies [4]. Another stressor linked to the sedentary way of life that is not always cited in archaeological research was the emergence of pests.

Most of the pests visible in the archaeological record are insects or rodents. Although there are certainly other species [5,6], this study only focuses on these two animal groups.

Delimiting the actual change from anthropophilic to commensal rodents and finally to pests is difficult to trace over time [7], and the evolution of certain insects into synanthropic or pest species is likewise not fully understood. However, it can be assumed that the domestication of plants and animals, agriculture, animal husbandry, and food storage practices for certain animal species (especially insects and rodents) led to new habitats linked to humans offering optimal living conditions [8]. Settlements with dwellings offered protection from predators or competitors as well as shelter from fluctuations of temperature, while storage structures ensured a constant, season-independent supply of food [9]. In addition to a permanent access to food, the adaptions of the grains themselves linked to domestication also offered a decisive advantage to pests, as they were larger and more nutrient-rich than their original wild counterparts [10]. These different factors thus probably led certain animal species to develop close ties with humans as pests.

Although there is evidence in different ecological and climatic contexts that insect pests accompanied the early farmers during their spread from southwest Asia through the Balkans towards central Europe (the so-called Danubian route of spread) [8], there is little or no information of this type for the maritime route towards the Iberian Peninsula, where the environmental and climatic conditions were presumably analogous. The crop spectrum in the north-western Mediterranean area was broader compared to that of central Europe, notably involving naked wheat [11], which is generally absent from sites along the Danubian axis. This crop is well adapted to the Mediterranean climate and was easily threshed and stored as clean grain, but it is also often attacked by insect pests, who fed from the grains without hulls. After a long period of stability of the crop assemblage in the area, certain authors suspect an influence of a series of stress factors after 4000 B.C., such as an increase of soil erosion due to anthropogenic or natural fires, global cooling, hydrological oscillations, and periodical flooding [12,13,14,15,16], coinciding with an apparent regional demographic decline (observed in southern France) [17,18]. These factors appear to have yielded an agricultural model where glume wheats gain in prominence [3,12]. Could pests have played a role in this change? To date, few western Mediterranean Neolithic studies shed light on the subject of insect or rodent pests. This is mainly due to the comparatively small number of sites benefiting from waterlogged preservation, as work has mainly focused on cave and open-air finds, where insect and small mammal finds are poorly preserved (or not preserved at all) and in relatively small numbers. For a summary of the Neolithic wells excavated so far in southern France, see Eric Thirault et al., 2014 [19]. Of these excavated wells, only a few such as the well of Clos de Roque in Saint-Maximin-la Sainte-Baume [20], have been the subject of entomological research. The same situation applies to research on small mammals. Furthermore, rodent remains, especially in caves, tend to be thought of as post-depositional intrusions often not directly related to human activity [21,22].

**Figure 1:**
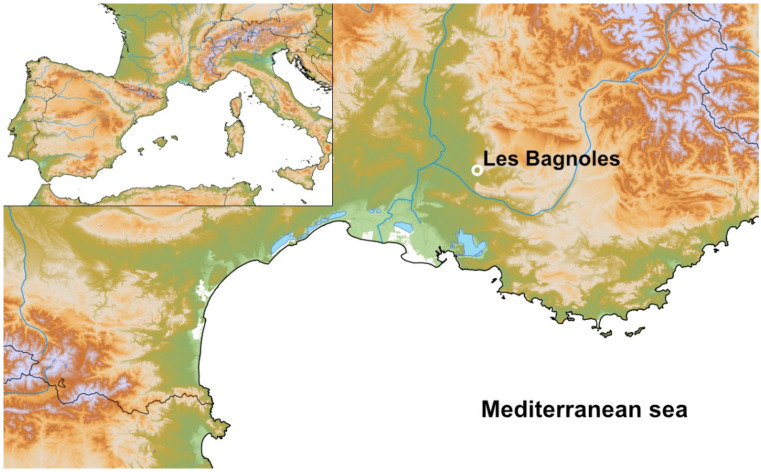
Map with the location of the archaeological site Les Bagnoles, L’Isle-sur-la-Sorgue, Vaucluse Department, SE France. (Software: QGIS3.6, © European Union, Copernicus Land Monitoring Service (2016), European Environment Agency (EEA)).

**Figure 2:**
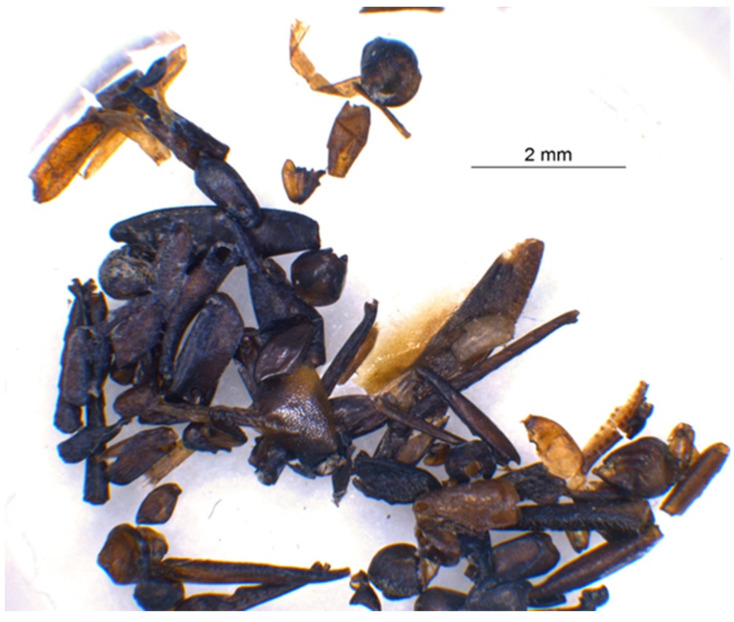
Examples of insect remains from well 990 that evidence the excellent conditions of preservation of organic material in the waterlogged layers. Photograph: Marguerita Schäfer.

The potential of identifying pest infestation by insects and rodents can therefore only take place at sites characterised by conditions of preservation favourable to organic remains. This is exactly the case of the open-air site of Les Bagnoles (L’Isle-sur-la-Sorgue, Vaucluse, Provence-Alpes-Côte d’Azur) in southern France [23] (Figure 1), featuring three Middle Neolithic water wells dating between 4250 and 3700 cal B.C. These wells with waterlogged deposits contain a rich and well-preserved assemblage of finds of insects and rodents (Figure 2). Furthermore, study of the plant remains from the three wells likewise has yielded compelling data on crop husbandry, agriculture development, and changes in crop cultivation during the Middle Neolithic. The plant findings also highlight a clear shift in the crop spectrum around 4000 cal B.C., a phenomenon identified elsewhere in southern France [3,12,24,25]. While the better-represented crops during the 5th millennium B.C. in the area were naked wheat (*Triticum* cf. *durum*) and naked barley (*Hordeum vulgare* var. *nudum*), after 4000 cal B.C., glume wheats gained importance, and einkorn (*Triticum monococcum*) became the main regional crop during the 4th millennium B.C. Although several hypotheses venture to explain this change [12], it has never been possible to investigate an assemblage of waterlogged finds containing pre- and post-harvest pests in the framework of farming systems where the pests could have acted as agents of change (for a more detailed description of this regional shift, see [12]).

This article presents the assemblage of insects and small mammals contained in the wells of Les Bagnoles, placing a particular focus on the potential and obligate pest species that could have damaged both standing and stored crops. The current paper does not intend to offer a detailed presentation and discussion of the entire microfauna of this site, in particular, the broad insect assemblage. However, preliminary results can be consulted in the monographic publication of the site [25], and the final results will be examined elsewhere and in connection with the study focusing on ecological aspects [26]. Furthermore, this article also investigates the strategies adopted at this Middle Neolithic site relative to changes in crop cultivation, crop choice, farming, and storage practices in response to pest infestation. Its findings therefore serve to discuss the potential pest management strategies applied in this early timeframe.

### 1.1. Prehistoric Crop Pests: An Overview

The initial definition of the term pest [27,28] appeared around 1880 in reference to *Phylloxera* (wine pest). It has since served most often to designate organisms that prompt economic damage [29]. A recent study on the pathogens and pests of major food crops (wheat, rice, maize, potato, soybean) [6] confirms that pests even today are a major obstacle to ensuring food security. Pests, in fact, reduce yearly worldwide wheat production by 21.5% (mean of 10.1–28.1%) [6]. This predicament, according to the FAO, is exacerbated by climate change [30]. Another study on global cereal harvests [5] estimates that pests trigger losses of 15%, of which 20% stem, respectively, from fungi, rodents, and birds, while the remaining 80% is linked to a variety of different insects.

This recent high loss rate and pest pressure can nonetheless hardly be compared with the conditions of the Neolithic, as ancient plant cultivation took place at a much smaller scale, and pests first had to make the transition from natural to new human-related habitats [31]. However, Neolithic crop production, as that of today, certainly attracted two groups of pests, notably pre-harvest pests that damaged the plants, fruits, and roots in the field and post-harvest pests that harmed stored products.

#### 1.1.1. Rodents

Pests throughout the world include three rodent suborders: Myomorpha (rats, mice, voles, hamsters, gerbils, jirds, mole rats), Scuiromorpha (squirrel-like rodents), and Hystricomorpha (porcupines, cane rats, and other, usually larger rodents) [32]. Of the approximately 2000 known species, probably less than 250 have evolved into pests [33]. The most common post-harvest pests are from the Muridae family. Pre-harvest pests, in turn, correspond mainly to species from the vole family (Arvicolidae) [34]. Additionally, rodents play an important role in the transmission and spreading of different diseases (e.g., plague, leptospirosis, leishmaniasis, salmonellosis, and viral haemorrhagic fevers) and are carriers of arthropod vectors and ectoparasites, such as ticks, mites, lice, and fleas [35].

Before the advent of agriculture in Neolithic times, rodents were probably already attracted to human food or waste. This is evidenced, for instance, by rodent gnawing marks on fruit stones from Upper Palaeolithic layers (15,000 B.P.) in the cave of Cova Fosca (Castelló, Spain) [36]. Moreover, the house mouse apparently accompanied the hunter-gatherer societies of the Natufian culture around 14,500 years ago as they gradually spread through the Levant theoretically prior to cultivating crops [7,9].

Although small mammal remains are regularly recorded at Neolithic sites in Europe, these types of finds have served, for the most part, as sources of information on environmental and climatic conditions [37,38,39,40]. Certain small mammals also were a source of food for ancient populations [41,42,43]. Studies on the potential of rodents as pests in praehistoric and historic times mainly refer to species introduced in southern, western, and northern Europe, such as the house mouse (*Mus musculus*), the house rat (*Rattus rattus*), and the brown rat (*Rattus norvegicus*).

Cucchi et al. [9,44,45] reconstructed the route of the house mouse from the eastern Mediterranean to Europe. Thus, the initial approach to humans by the house mouse appears to have taken place in the Levant about 14,500 years ago. This rodent then spread throughout the Middle East in parallel with the early farming cultures about 12,000 years ago before arriving 10,800 years ago on the Island of Cyprus. Its spread appears to have slowed down drastically after that. It was only during the development of proto-urbanism and exchange networks when the house mouse spread further into eastern Europe 6500 years back and into southern Europe 4000 years ago before establishing itself at a large scale. Colonisation of the entire western Mediterranean Basin and northern Europe by the house mouse is assumed to have taken place only throughout the 1st millennium B.C. [44]. Other studies reveal that the house rat and the brown rat spread in Europe later than the house mouse [46,47]. In any case, the history of the distribution of these three rodent species clearly indicates that they were not autochthonous to Europe and acted as pests long before their arrival.

This raises the question of whether other Pre-Neolithic rodents in Europe, prior to the arrival of the exogenous species, already played a role as commensals or pests. The analyses of rodents from the Neolithic site Skara Brae (Orkney) suggests that endemic voles were subjected to pest control while likewise serving as a source of food [42]. Otherwise, rodents are hardly cited as field pests in prehistoric times although finds of voles appear regularly at archaeological sites throughout Europe [40,48,49,50]. Wood mice, representatives of the genus *Apodemus*, are also common throughout European sites. The most frequent species are the common wood mouse (*Apodemus sylvaticus*) and the yellow-necked mouse (*Apodemus flavicollis*), whose role as pests in past times is hardly discussed in detail.

This study places particular emphasis on the species *Apodemus sylvaticus* (wood mouse), which is currently one of the most common mammals in Europe. The species has been known since the Pliocene [51,52] in the area extending between north-western Africa and Europe to the Ukraine and Belarus. It was likewise introduced by humans into the British Isles, the different Mediterranean islands, the Channel Islands, and the islands of the Atlantic [48,53]. This species lives in deciduous and mixed forests, forest edges, hedges, gardens, parks, as well as in fields and meadows, from where it often visits buildings in winter [54]. Its diet consists mainly of seeds and fruits, nuts, buds, mushrooms, root tubers, and insects. Since it is very adaptable depending on the availability of plant food, it is considered a pioneer species [55]. It can be assumed that the wood mouse as well as other *Apodemus* species occupied the commensal niche of human settlements before the arrival of the house mouse [9]. Remains of yellow-necked mice from a granary at the Late Neolithic site of Chalain 3 in eastern France appear to support the notion that *Apodemus* species was commensal [48]. Furthermore, a long-term experiment on Late Neolithic forest-field cultivation has revealed that both voles and wood mice can damage field crops [56]. Today, the wood mouse is usually considered commensal to humans and a storage rather than a field pest.

#### 1.1.2. Invertebrates

Numerous archaeoentomological studies offer evidence of the early occurrence of various pre- and post-harvest invertebrate pests. Two of the most important insect pests are discussed below. The grain weevil (*Sitophilus granarius* L.) and its two relatives (*Sitophilus oryzae* L. and *Sitophilus zeamais* M.) are, according to current research, the first storage pests to feed on prehistoric crop stores and to colonise the new habitats [57,58,59]. The geographical origin of the grain weevil has yet to be clearly identified. It probably originated in the forests of the Near East [60]. Nowadays, it has a cosmopolitan distribution, making it one of the most feared storage pests. However, it provokes the most damage in temperate zones, as its optimal temperature range is 26–30° [58].

G.A. King mentioned a possible indicator of insect crop pests for the Natufian culture (*c*. 13,000 B.P.). He pointed out holes on certain charred grains of wild einkorn at the site of Abu Hureyra, Syria, which could be regarded as feeding marks of grain weevils [58]. However, after King, this classification remains vague, as these types of structures can also stem from gas bubbles during grain carbonisation.

The earliest secure traces of the grain weevil include finds from a PPNC well at Atlit-Yam (Israel) dated to *c*. 6250 B.C. [61], Dispilio (Greece) dated to *c*. 5700 B.C. [62], and various Linear Pottery Culture wells in Germany, such as at Leipzig-Plaussig, dated to *c*. 5250 cal B.C. [63,64]. A grain weevil was also present in a dog coprolite collected in a Middle Neolithic stratum at the Arene Candide cave (Italy) dated to *c*. 5000–4200 B.C. [65].

The rapid spread of the grain weevil during the Early Neolithic is remarkable, as it is unable to fly and only reaches a maximum size of 3.7 mm [66]. This means that it dispersed passively, hand in hand, with the grain from its origin in the Near East as the Neolithic advanced throughout Europe. Beetle infestation of cereals can in fact remain undetected for a long time due to the small size of the grain weevil and its reproductive behaviour. Grain weevil females, in fact, form breeding nests in grain stores and increase the ambient temperature and humidity in infested grain stocks by their movement [63]. Their softening of the grain kernels, coupled with an increase in humidity, often provokes mould, a secondary damage to the grain. The females bore a hole into the soft cereal grains before laying an egg and sealing the opening rendering the infestation invisible. This is followed by the development in the grain of the egg into an imago. Only the husk remains after the beetle hatches about five weeks later. Furthermore, young beetles become sexually mature and can successfully reproduce again only after a few days depending on the food supply and the ambient temperature. Under optimal conditions, up to three generations can hatch per year. Although they mainly attack wheat, barley, rye, and oats, they also feed on acorns and buckwheat. Legumes, in turn, are poisonous for grain weevils [5,67,68].

What is unusual is not only the rapid spread of the granary weevil in Central Europe during the Early Neolithic but its absence in the wetland settlements of this zone during the Late Neolithic [62,69]. Why it disappeared again after its rapid spread during Central Europe’s linear pottery culture remains unclear. It is possible that the human populations changed their grain storage methods and thus provisionally halted its spread. In fact, the earliest published grain weevil in France so far is from a Late Bronze Age settlement at Lac du Bourget (Savoie) dating to *c*. 1100–900 B.C. [70], a site that is only about 270 km north of Les Bagnoles.

Various field pests have likewise been recorded in Neolithic contexts elsewhere in Europe. Examples of the seed beetle (Bruchinae) group are also common. An example is the pea beetle (*Bruchus pisorum* L.) from Layer 3 of the Horgen culture lakeside settlement of Zürich-Parkhaus Opera dated to *c*. 3176–3153 cal B.C. [71]. A compilation of the different sites with finds of synanthropic species or pests is included, for example, in the different studies by Panagiotakopulu and Buckland [8] and King et al. [58].

## 2. Materials and Methods

### 2.1. The Archaeological Site of Les Bagnoles, Its Three Middle Neolithic Wells (Structures 250, 990, 994), and Their Contexts

The site of Les Bagnoles is located in the south of the Vaucluse Department in the Provence-Alpes-Côte d’Azur region about 16 km east-south-east of the city of Avignon and 2.5 km south-west of L’Isle-sur-la-Sorgue at 57 m.a.s.l. (Figure 1) [23]. The excavation (1.5 hectares) took place in the alluvial plain of the Durance and Calavon Rivers towards the centre of the agricultural Plain of Comtat. The site has been known since 2006, when an archaeological prospection revealed burial structures and other materials [72]. A research excavation followed from 2012 to 2015, carried out by several partner institutions (University of Basel, University of Aix-Marseille; Service Régional de l’Archéologie de Provence-Alpes-Côte d’Azur and the Direction du Patrimoine de L’Isle-sur-la-Sorgue). An archaeological evaluation, as well as the first results of the archaeobiological investigations, was published in 2020 in the monograph coordinated by Samuel van Willigen [23,24,25]. In addition to the occupation from the Middle Neolithic (Néolithique moyen), the site also yielded structures from the Bronze Age, Iron Age, Gallo-Roman, and Modern periods, which will not be treated in this article. The Middle Neolithic features of Les Bagnoles were mainly sunken structures, such as pits, silos, and, key to this study, wells (Strs. 250, 990, and 994), ranging between 4250 and 3700 cal B.C.

Well 250, dating to 4250–4050 cal B.C., is the oldest of the three [73]. It is 2.50 m deep (Figure S1a) and is characterised by an upper section with a bowl-like depression (3 m in diameter and 60 cm deep), which narrowed into an off-centre vertical cylinder below along its eastern half. The assemblage of pottery in its fill falls in line with the “Chassey” Middle Neolithic tradition. The archaeobotanical and large mammal remains contained in well 250 reveal that activities related to the preparation and consumption of food were carried out in the immediate vicinity of the excavated structures [74]. Furthermore, the plant remains are compatible with the local vegetation of the time [12,24,75].

Another 72 structures from this timeframe are located in what appear to be shallow natural hollows probably linked to the site’s natural topography. The area of this older phase formed part of an active alluvial plain-type environment [15]. Furthermore, the excavation revealed a large hollow filled with heating stones, a possible silo pit whose dating remains unclear, cremation graves, and several mortuary structures [15,24]. There is so far no indication of the presence of buildings that can be attributed to this occupation [15]. However, the numerous shallow structures, as well as other larger and deeper features, suggests that the absence of underground silos cannot be attributed to erosion. It is also possible, due to the absence of the original ground level, that the dwelling features are today archaeologically invisible. Therefore, a reconstruction of the function and the organisation of this space is difficult. For the moment there are two main hypotheses: The first supposes it corresponds to “a zone of activities linked to a nearby habitat, with a nearby peripheral area relegated to the dead” [15], while the second points to “a link between the graves and these same zones of activities used probably for ritual purposes (feasting?)” [15].

The calibrations of the dates of the other two wells, Str. 990 (4050–3980 cal B.C.) and Str. 994 (3950–3750 cal B.C.), place them in a later phase of the Middle Neolithic (Figure S1b,c) [12,73,76]. Moreover, its ”La Roberte”-type pottery places it in the Middle Neolithic.

Well 990 comprises a roundish-rectangular upper section (1 m wide and a max. depth of 40 cm) before narrowing into cylindrical shape attaining a depth of 3.30 m. The upper section of well 994, with a max. depth of 3.10 m, is also roundish-rectangular before assuming a bell shape (probably due to a temporary rise of the groundwater level) before becoming round-rectangular. Forty-two other structures of this timeframe were unearthed apart from these two wells. They include small and large pits as well as two underground silos. This phase also reveals no trace of dwellings and, unlike the earlier Middle Neolithic sequence, it has no burials. However, the presence of structures potentially linked to storage (round pits and silo pits) suggest settlement activities [25].

### 2.2. The Fill of the Wells, Preservation, and Taphonomical Aspects

As the three wells lacked any sort of casing, it can be assumed that their lifespans were limited. The three were presumably constructed within a short timeframe, perhaps during a brief, yet extreme drought during the annual dry summer season, as this alluvial region is hardly characterised by water shortage [23]. Wells are nevertheless characteristic of farming sites and have been identified in many Neolithic contexts, particularly in Central Europe [77,78]. Furthermore, the ^14^C datings suggest that after their use, they were probably backfilled within a span of a few years to a few decades [73].

**Figure 3:**
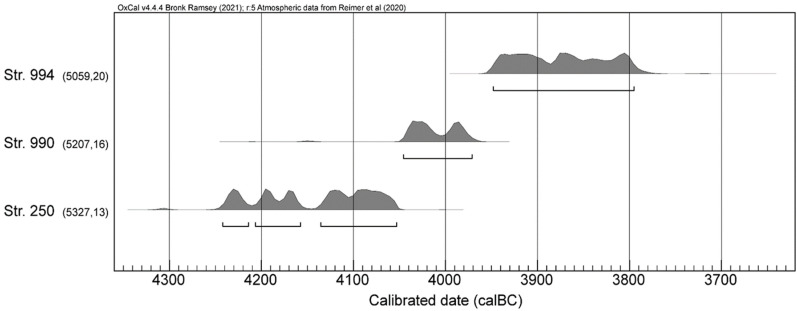
Single plots of the contemporaneity test results for the well 250, 990, and 994. Calibration with OxCal v4.4.4 [80]) and IntCal20 atmospheric curve [81].

The stratigraphical sequence of the wells is only approximately known (Figure 3) for the upper levels that were preserved in dry conditions. In the waterlogged part at the bottom of the wells, excavating was extremely difficult. These levels were excavated by means of artificial spits ranging between 20 to 10 cm in thickness [12] (Figure S1a–c). The finds examined in this paper come from samples gathered among the lowermost levels of the three wells and were described by semi-quantifying preservation indicators as published in previous research [79]. For further clarity, a summary of these descriptions was added to the supplementary data Table S1.

Well 250 contained numerous mollusc and bone remains (including calcined bone fragments) were identified throughout the sequence. Yet, indicators for permanent and good waterlogged preservation conditions were observed only in the lowest levels (samples 38–42 from the northern half of the well and samples 73–74 from the southern half (Table S1) although these levels also contained many roots suggesting a degree of bioturbation. The insects and small vertebrate remains were collected for the most part towards the base where molluscs were less abundant. This could be interpreted as a rapid anthropogenic filling of the well’s base by means of daily discharges of waste (cereal chaff remains, dung, pottery, etc.).

The lower, waterlogged fill of well 990 (and to a less degree that of well 994) consisted of a highly organic and well-preserved deposit comprising mostly animal dung [12] and abundant insect and small vertebrate remains [25]. Indicators of root bioturbation were less common in the dung-rich samples of well 990 (samples 69–74). Molluscs were also rare. By contrast, they were very abundant in well 994, where the indicators of preservation suggest a quality that was not as optimal. 

This permanently humid atmosphere conserved in the lower levels of well 990 and 994 a large number of plants, pottery, stone tools, and large animal bones. The plant spectrum and the large animal remains from these lower, waterlogged levels again mainly corresponds to settlement waste (for more details about formation processes of cultural depositions in wells, see [16], ch. 3.4.2.1, pp. 66–67).

### 2.3. Archaeozoological Material and Analysis

The current analysis is based on an assemblage of small mammals and invertebrate remains recovered from multipurpose sediment samples in the lower, waterlogged section of the fills of the three wells. Each sample consisted of up to eleven buckets (about 8 L each) of sediment. Not the whole sediment was processed but often a large part of it (see Table S1). The small mammal and invertebrate finds were extracted by wet sieving using the very gentle wash-over method in order to preserve/save all the very fragile remains [82] with meshes of different sizes (4, 1, and 0.35 mm) [83]. This method aimed at washing the sediment and separating the organic material including plant and invertebrates remains from the inorganic material, which contained most of the small animal bones.

To recover the small mammal bones, the inorganic 4 mm and 1 mm fractions from 18 samples (consisting of selected 71 buckets and 588.3 L of sediment, see Table S1) were sorted by stereo microscope (6–40× magnification). A few small animal remains identified during sorting of the botanical remains from the organic fraction were handed over by the archaeobotanist to us and included in the small mammal study. The remains were identified by referencing the osteological collection of the IPAS and other relevant keys [21,49]. Biometrical analyses of teeth undertaken with the measurement software (IMAGE J) following the Pasquier 1974 [84] procedure served to distinguish between the different *Apodemus* species. The information as to the small mammals was introduced into Ossobook, a software database specially designed to store and process archaeozoological content [85]. In addition to several plant finds [12,73] from the waterlogged sections of the wells, the remains of three mice from wells 990 and 994 were ^14^C-dated.

All invertebrate remains were selected from the organic fractions of the sediment samples during the analysis of the archaeobotanical remains [22] and retained for the invertebrate analysis. A total of 31 samples were examined for invertebrate remains. Table S1 lists the sediment samples, their volumes, and the examined volumes as well as the origin of the different invertebrate fractions. The organic 4 mm (when available) and 1 mm fractions were sorted entirely. The 0.35 mm fractions were subsampled with the grid method [12,86], and *c*. 10 mL of residues were sorted. For further explanation as to the sediment sampling on excavation and sample processing during the archaeobotanical analysis, see [12,15,75]. The invertebrate remains were placed in distilled water during processing and then stored in ethanol. They were then subjected to stereo microscope analyses (6–100× magnification) and identified by consulting the entomological collection of the Natural History Museum Basel. Following the archaeobotanical procedure [12], the number of invertebrate remains from the subsamples of the 0.35 mm fraction was also multiplied to estimate the values for the total volume of the fractions, a procedure commonly done in archaeobotanical research [86,87].

## 3. Results

### 3.1. Radiocarbon Dating

Wells 250, 990, and 994 benefitted from a total of 14 radiocarbon analyses (Table 1, Figure 3 and Figure S2) [12,73]. In the framework of the current study, three Apodemus/Muridae bones were dated in order to prove that they were not recent intrusions. Sample ETH-113018 from well 990 yielded 5161 ± 33 BP, a range that although roughly contemporary is slightly younger than those obtained from charred cereal grains. However, the C/N ratios are out of expected range; thus, the result has to be considered with caution. Samples ETH-113016 and ETH-113017 equally yielded old dates (Figure S2). They are, in fact, older than most other dates obtained from this well. Notwithstanding, the findings confirm the ancient origin of the bones. Regrettably, the small size of the bones resulted in an amount of carbon lower than 0.5 mg. Hence, the results obtained from such small bone must be viewed with care. In any case, for the purposes of this paper, the results bolster the assumption that the finds are most likely contemporary to the other materials in the fills of the wells. Furthermore, considering all these characteristics and benefitting from several dates for each structure, a contemporaneity test was undertaken to verify their statistical homogeneity [80,88]. This allowed to blend the different dates into a single value (Figure S3) to more adjusted chronological calibrated ranges (Figure 3), notably 4236–4057 cal B.C. for well 250, 4046–3972 cal B.C. for well 990, and 3949–3796 cal B.C. for well 994.

### 3.2. Microvertebrates

A total of 1907 small mammal remains were retrieved from 18 samples, of which 984 could be identified to the rank of order, genus, or species (Table 2). The remains of the small mammals consist of 62% complete skeletal elements. The number of other micro-vertebrates, in turn, is low (herpetofauna *n* = 151; fish *n* = 23; bird *n* = 9). The dark discolouration of the remains of wells 990 and 994 is similar to that identified at lakeside dwellings. It is probably linked to the humic acid of the highly organic environment due to the presence of dung. Otherwise, traces of burning (*n* = 8), digestion (*n* = 3), or butchering (*n* = 0) were rare or absent.

**Figure 4:**
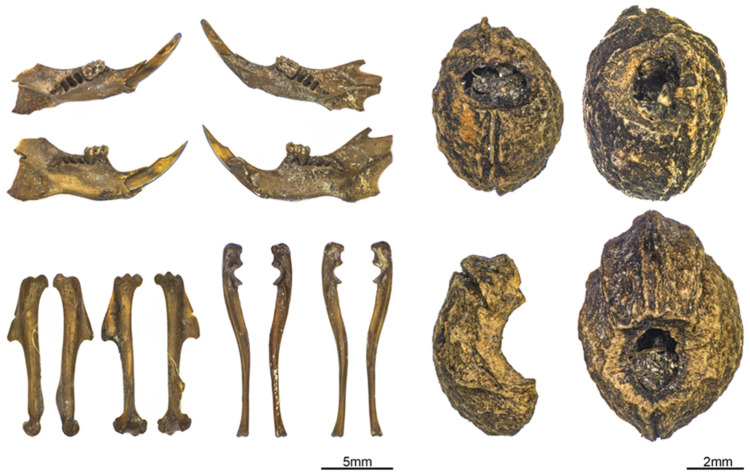
(**Left**) Selection of *Apodemus* cf. *sylvaticus* remains from well 990 and 994. (**Right**) Blackthorn fruit stones (*Prunus spinosa*) with mice gnaw marks from well 250. Photographs: Raül Soteras.

The small mammal remains correspond to three orders: Carnivora (*n* = 60), Eulipotyphla (*n* = 17), and Rodentia (*n* = 907). The majority are Rodentia, represented most often by murid remains (*n* = 410, MNI = 47) assigned to the genus *Apodemus* (Figure 4). The genus *Mus*, which from the morphological point of view is similar to the *Apodemus*, was not identified. The two genera as well as the representative species are nonetheless easily distinguished based on the size of their skeletal elements and tooth morphology [21,89].

**Figure 5:**
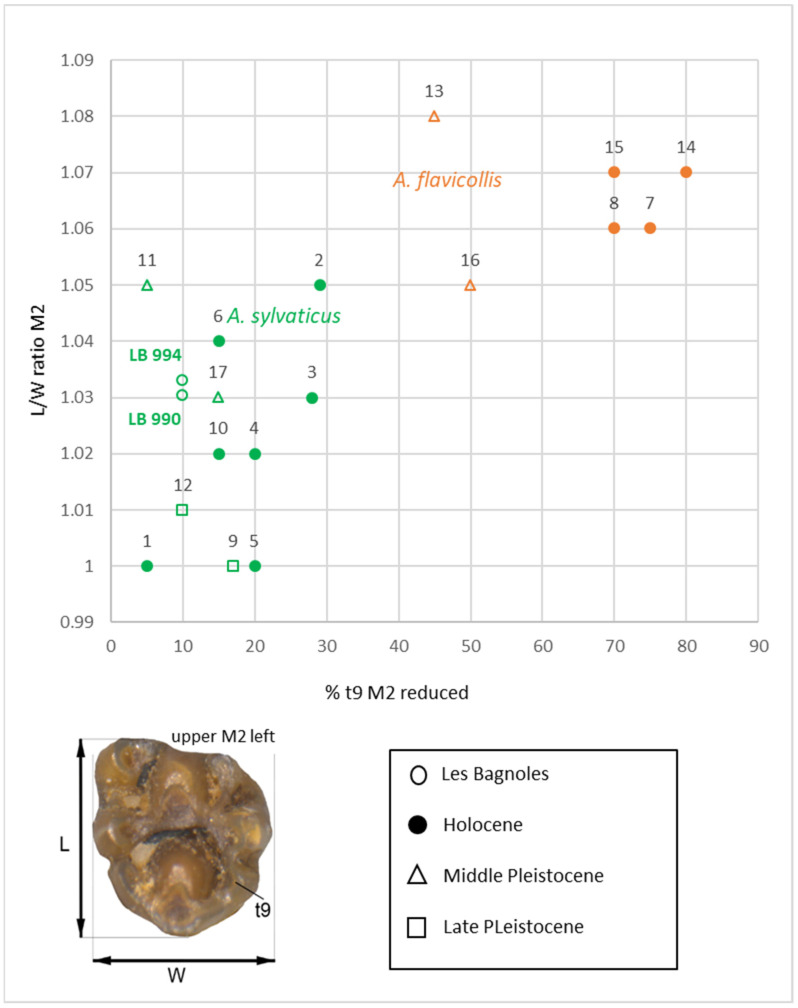
*Apodemus* finds: Scatterplot comparing L/W ratio of upper M2 and percentage of specimens with reduced t9 (tubercle 9); L 990: Les Bagnoles, well 990; L 994: Les Bagnoles, well 994; 1: Northern France, 2: Le Claux, 3: St. Mathieu de Tréviers, 4: Camargue, 5: Burgos, 6: Kirchdorf, 7: France, 8: Kirchdorf, 9: Santeney, 10: Combe-Grenal, 11: Grimaldi, 12: Le Lazaret, 13: Cendres Cave, 14: Cueva del Agua, 15: Las Yedras, 16: El Higuerón, 17: Castillejo del Bonete. 1–16 after [84,89]. Photograph: Raül Soteras.

Three naturally occurring species of the genus *Apodemus* (*A. sylvaticus*, *A. flavicollis,* and *A. alpinus*) in the region of Provence-Alpes-Côte d’Azur (Vaucluse) [90] come into question for the Apodemus remains from Les Bagnoles. The species *A. alpicola* can largely be excluded, as it is distributed exclusively in the alpine regions of Provence-Alpes-Côte d’Azur [90]. While *A. sylvaticus* is most widespread, *A. flavicollis* is currently found in the north of the Vaucluse Department. Little is known as to a possible shift of their regional sphere of distribution in the past [90]. The two species are very similar in appearance, behaviour, and biotope requirements, and they coincide as food competitors in certain regions [56]. Moreover, it is hardly possible to differentiate the two species based on their postcranial skeletons. The method of differentiation from their tooth morphology advanced by Pasquier [84] relies on morphometric analyses of the second upper molar (length/width ratio) and the level of development of its tubercle t9. Our analysis of the *Apodemus* teeth indicates that most (90%) of the second upper molars of wells 990 and 994 have a well-developed t9 (tubercle 9) and length/wide ratios of about 1.03 (well 990) and 1.033 (well 994), complying with the variability displayed by both modern and fossil *Apodemus sylvaticus* assemblages (Figure 5). The results therefore suggest *Apodemus sylvaticus* to be the most represented species in the wells as well as the most often detected in the Vaucluse Department today.

**Figure 6:**
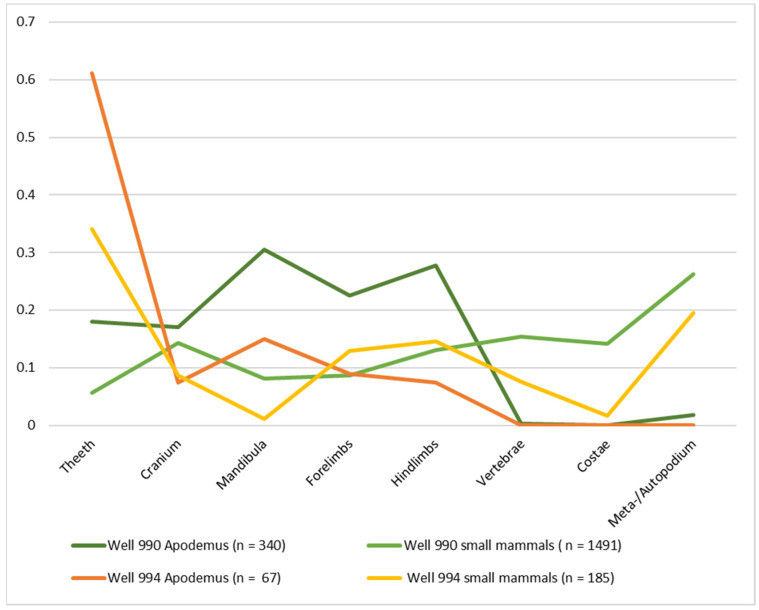
Proportions (%) of cranium, mandibula, isolated teeth and postcranial body parts of *Apodemus* cf. *sylvaticus* and of combined small mammal categories Muridae/Arvicolidae and small mammals indet. from wells 990 and 994.

Figure 6 illustrates that it was possible to identify molars, skull bones, and long bones of the forelimbs of wood mice. However, it is also possible to assume the presence of other skeletal elements of *Apodemus* sp. of the murid/vole (Muridae/Arvicolidae) category as well as undetermined small mammals. These include skeletal elements such as meta- and autopodium (feet and hand bones; finger and toe bones), vertebrae, ribs, and loose incisors that cannot be determined accurately but fit in terms of size. It is likewise difficult to differentiate certain fragmented hindlimb bones (especially femurs and tibias) and bones of young murids and voles, as it is not possible to pinpoint their key morphological criteria [49,91]. The certified distribution of skeletal elements, coupled with the fact that hardly any remains of other rodent species were detected among the finds, suggests that the cases that cannot be further identified correspond at least in part to *Apodemus* sp.

The composition of the skeletal parts indicate that the animals entered the wells whole and not as specific body parts. Estimates of the age of the 57 individuals from well 990 based on observations of the epiphyseal closure of their long bones [44] point to 15 adults, 30 subadults, and 12 juveniles or infants.

The vole remains comprise two species, notably the Mediterranean pine vole (*Microtus duodecimcostatus*) and field vole (*Microtus agrestis*), which are still present in Vaucluse Department [92,93]. The Eulipotyphla order consists of five remains of shrew (*Crocidura russula*) and mole (*Talpa* spec.). Although the skeletal elements (two phalanges 3 and a single M2) of wells 250 and 990 cannot be identified at the species level, they correspond presumably to *Talpa europaea* and not to *Talpa caeca*. These in France (at least currently) are known only in a very isolated zone near the Italian border and otherwise only further east and at higher altitudes, in Italy, Switzerland, Greece, and Bosnia-Herzegovina [94]. The remains of the order Carnivora, all from well 990, are mustelids. With the exception of two (from sample 71), all are from sample 70 and presumably belong to a single individual (either a *Mustela nivalis* or *Mustela ereminea*). The intraspecific size variability is high in both species, and their long bones can hardly be distinguished morphologically [95,96,97]. Only two skeletal elements (a bacculum and a mandibula) are identified as *Mustela nivalis*. Otherwise, among the hand-collected materials from wells 250 and 990, there are other bones of each of the mustelid species [25].

The study of the density of the finds indicates that different quantities of small mammals found their way into the three wells. While in well 250, only 52 remains (density of 0.7 per litre of sample sediment) were found, well 990 contained 1648 remains (density of 4.7). The finds in well 994 total to 203 remains (density of 1.2). Overall, the well samples reveal significant differences in proportion. Furthermore, they demonstrate a very low diversity of species and a clear dominance of *Apodemus* *sylvaticus* cf. As the number of finds of well 250 was low, no MNI could be established. Those of wood mouse in well 990 (*n* = 340; MNI of 42) are by far the most abundant (Table 2). It is noteworthy that these remains were mainly collected in samples 70 and 71. Sample 70 comprised a total of 537 small mammals remains (including 195 remains from wood mice), while sample 71 had 230 (111 of wood mice). The other samples contained ≤20 remains (≤12 of wood mice) in each case. Small mammal as well wood mice remains in well 994 (*n* = 67; MNI of 4) are less plentiful than in well 990. Most of them appear in samples 41 (*n* = 87, with 37 wood mice) and 42 (*n* =107 with 20 wood mice), while they were almost absent in the other two samples (<10 remains).

### 3.3. Invertebrates

The excellent conservation of the lower waterlogged layers of the wells led to the recovery of a total of 10,778 invertebrate remains, which, depending on the sample volume, corresponds to a (extrapolated) total of 23,392 fragments (Table 3). All of the following figures listed below in brackets represent the extrapolated values. Most of the insect fragments are from beetle species, embodied in the three wells by 17 families. Open wells, in fact, can function like large barber traps for ground-dwelling beetles, which explains their high proportion [98,99]. Entomological studies of the waterlogged deposits of other wells reveal the same pattern [100].

Well 250 yielded a total of 1515 (7699) remains. Of them, 1088 (4472) are fragments of beetles, of which 255 (644) can be assigned to the rank of species and 399 (1552) to either family or genus. Well 990 contained 2386 (4168) invertebrate remains. The majority consisting of 1910 (3485) are assigned to beetles, of which 371 (504) are species, and 1139 (1936) either family or genus. Well 994 alone held 6877 remains (11,526), including 3811 (5910) fragments of beetles. Of these, 591 (744) could be assigned at the rank of species and a further 1046 (1690) to either family or genus.

**Figure 7:**
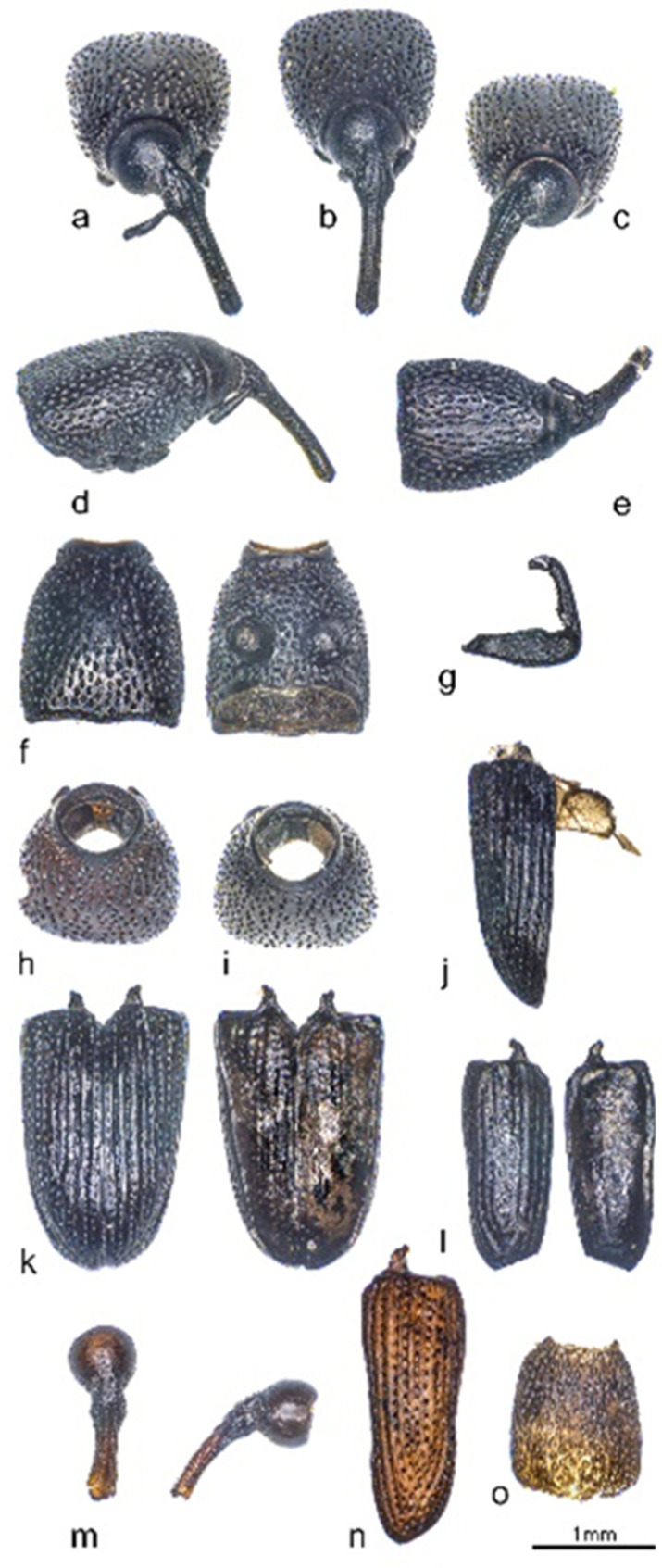
Selection of skeletal elements from *Sitophilus granaries*; (**a**–**e**) well 990, head and pronotum; (**f**) well 990, pronotum; (**g**) well 250, extremity; (**h**) well 994, pronotum; (**i**) well 990, pronotum; (**j**) well 990, elytron; (**k**) well 994, elytra; (**l**) well 250, elytron; (**m**) well 994, head and pronotum; (**n**) well 994, elytron; and (**o**) well 994, pronotum. Photographs: Raül Soteras.

The preservation of the organic remains of well 250 is on the whole worse than that of the two later wells. Among the specific Coloptera fragments (species to family) of all three wells, those of the Scarabaeidae are clearly dominant. In well 250, it corresponds to 92.5% (77.7%), while in wells 990 and 994, its amounts are 90.8% (86.2%) and 73.2% (74.5%). Scarabaeidae include real dung beetles, such as *Gymnopleurus sturmi*, now very rare in southern France, and small dung beetles, such as *Calamosterus granarius* [101]. Aquatic invertebrates, in turn, are only represented by a few finds in wells 250 and 990. All the aquatic insect larvae remains were found in the deepest 60 cm of well 250 and in the lower layers of well 990. Moss mites, associated with moist environments, are also poorly represented in the three wells. The following discussion on the grain weevil resorts to MNI (estimated from the number of heads and pronota). Their highest value, 11 heads, is in well 990. The grain weevil (*Sitophilus granarius* L.), a primary storage pest, was detected, albeit in modest numbers (Figure 7), in all three wells. Well 250 contained six (pronotum) fragments, and well 994 had four heads.

Potential field pests were also identified among the finds of wells 990 and 994. Those of well 990 correspond to a wing cover (elytron) of a seed beetle. Since the wing cover could not be clearly determined, it was measured and compared with seed beetles species occurring in the western Mediterranean region and correspond most probably to the aspea beetle (*Bruchus pisorum* L.) or field bean beetle (*Bruchus rufimanus* B.). Both species, which are native to southern France, feed on legumes such as peas (*Pisum sativum* L.), field beans (*Vica faba* L.), and lentils (*Lens culinaris* M.) as well as flat peas (*Lathyrus* sp.) and vetches (*Vicia* sp.) [102].

Well 994 also yielded four elytra of ground beetles (*Zabrus tenebrioides* G). Although this beetle, which is widespread in Europe, was once a serious menace to winter cereals, it is no longer a threat in Europe [103,104]. The ground beetle has so far been recorded in certain Roman wells, such as Empingham (U.K.) [105] and Lynch Farm 2 in Orton Longueville, Peterborough (U.K.) [106], and Lattes-Saint-Sauveur (F) [107].

## 4. Discussion

The results of this study clearly indicate that both potential and obligate crop pests were present during the whole occupation of the site of Les Bagnoles. Although the distribution of small mammal and insect remains differs from well to well, the grain weevil is common to all three and suggests a long-term grain storage issue at Les Bagnoles. Moreover, wells 990 and 994, respectively, contained seed beetles and ground beetles, which serve as evidence of potential pre- and post-harvest insect pests. Wood mice were likewise increasingly present at least in the more recent Middle Neolithic phase.

Considering the permanent waterlogged conditions of the wells and the absence of traces of burrows, it is unlikely that the presence of these small animals can be interpreted as recent intrusions. Furthermore, the depth of the mammal finds (*c*. 3 m under the current ground level) does not line up with the burrowing depths of wood mice or other murids [108]. Although the ^14^C ages of the mice from well 990 reveal C/N ratios below the ideal (requiring caution as to how to interpret them in relation to the other more coherent C/N ratios), it is indisputable that these datings suggest a contemporaneity of the mice with the crops, thus bolstering the notion that the microfaunal elements are not recent post-depositional intrusions. Therefore, both the mice and insect remains contained in these features as well as the plants, large animal bones, animal dung, pottery, grinding stones, etc., are indicative of the temporal and spatial proximity of the Middle Neolithic occupation [12,75].

Wood mice were thus presumably attracted by the abundance of food at the settlement. Cucchi et al. [43] pointed out that the *Apodemus* is regarded as a commensal species as early as the Neolithic despite the rarity of archaeozoological evidence. In any case, the Neolithic assemblage of Les Bagnoles seem to confirm this assumption. Furthermore, regarding the history of distribution of the house mouse, this appears to confirm that wood mice occupied this niche in the settlements before being gradually displaced by the invasive house mouse [44,45].

Although the number of samples examined differs from well to well, the differences in density and number of finds as well as the accumulation of small mammal finds in certain samples of well 990 and 994 allow to advance compelling hypotheses. For the sparse evidence of small mammal remains and wood mice in well 250, it can be speculated whether there is a connection with the presumably non-sedentary function of the older occupation. This is nonetheless difficult to assess due to the unclear archaeological background and unclear origin of the well’s fill. The great number of wood mice remains in well 990 leads to further consider this animal as not only as commensal but also as a (storage) pest. This likewise raises the question whether its presence serves as a sign of a pest outbreak. Although wood mice are the most frequent in well 990, they are also common in well 994 (yet significantly lower in number, density, and MNI). In addition to their high number in well 990, there are other arguments that suggests that the vast differences in the quantity of remains do most probably not stem from taphonomic effects: The preservation of plant and invertebrate remains is excellent in wells 990 and 994 and, to a lesser extent, in well 250. Furthermore, the bone preservation of the hand-picked specimens and the remains from the samples (wet sieving technique, using wash-over) collected from the lower, water-logged layers of all three wells range from good to very good [24,25]. It is therefore possible to assume that these lower (waterlogged) layers offer a realistic portrait of the material that ended up in the abandoned wells when they were backfilled and what remained inside until they were excavated. However, it is not possible to reconstruct whether the rodent remains were disposed of directly in the abandoned wells or whether they arrived together with other materials (dung, straw, grains). However, since wood mice are concentrated in samples 70 and 71 of well 990, and as it is reasonable to assume that its backfilling took place over a short time based on the homogeneous dates of the different waterlogged layers [73], it is unlikely to assume an accidental entry of this great number of individuals over a short time span. One cannot exclude nonetheless that these wells acted as pitfall traps for many of the small mammals and other small vertebrates from the settlement’s surroundings [45]. The high numbers of a single species (wood mouse) and the low occurrence of other small animal species in wells 990 and 994 thus renders this scenario unlikely. Additionally, wood mice are very agile jumpers and can scale vertically [54]. It is therefore difficult to imagine that such a high number of individuals ended up inadvertently in the well’s archaeological layers. The “pitfall scenario” can nonetheless be retained for well 250 from the earlier Middle Neolithic phase due to the scarcity of the remains and their more regular presence throughout all the samples. On the other hand, the concentrations of remains in only a few samples of well 990 (and this can likewise possibly be assumed for well 994) suggest the presence of a larger and therefore more hazardous population of wood mice during a certain time span of the later Middle Neolithic settlement. This compares with the relatively high number of small mammals identified in a Roman well at the *vicus* of Studen Petinesca in Switzerland [50]. Archaeozoological analyses of the Roman materials at Petinesca suggest the well served as a waste pit for fur, leather, and horn industrial refuse as well as for more than 1000 mice remains (including 11 house mice and 97 wood mice). These are interpreted as the discarded carcasses of unwelcome inhabitants of the settlement [50], a type of scenario that can serve to explain the finds of wood mice at Les Bagnoles.

Today’s acyclic wood mice population fluctuations depend on food supply. Those in fattening years can rise to over 50 individuals per hectare, whereas in years with little access to food, they can decrease to below one individual per hectare [109]. The MNI of 42 in well 990 and the presence of different age groups also points to a healthy population. Furthermore, wood mice have been observed in human dwellings or stables, especially in winter, as they do not hibernate and require abundant sources of food and protection from the cold [54,55,68]. The remains of well 990 potentially serve as evidence that wood mice were more frequent at Les Bagnoles in the winter, attracted by a rich supply of food. As wood mice in regions of the Mediterranean reproduce even during winter, the presence of both adults and young in the wells is not surprising.

Finally, sloe stones in well 250 bearing traces of gnawing (Figure 4) as well as mouse coprolites collected in all three wells indicate that rodents competed with the inhabitants of Les Bagnoles for food throughout each of the Middle Neolithic phases. Even if it is known that wood mice are also pre-harvest pests [56], and the location of the well within the settlement, the circumstances of the finds, and the fact that apart from the remains of *Apodemus*, are also known, there are hence few traces of other field pests (especially voles); let us assume that wood mice from the wells targeted stored goods and perhaps settlement waste. Yet, the extent of the wood mice population and their threat cannot be fully defined. Did it attain a point requiring them to be decimated and disposed of? In any case, the option of eliminating them appears obvious when considering the great number remains in well 990.

The fact that insects, apart from mice, were also a problem for the residents of Les Bagnoles is demonstrated beyond all doubt by the presence of the grain weevil, the main storage pest, in all three wells. Although its numbers are modest in each of the wells, its persistence does not suggest a single event but a long-term grain storage issue. According to the current state of research, the *Sitophilus granarius* finds from the three Middle Neolithic wells, along with those from the Middle Neolithic layer of the Cave of Arene Candide (Liguria, Italy) dating to *c*. 5000–4200 B.C., are among the earliest records of its presence in the western Mediterranean [65]. Thus far, the earliest evidence in the current study area is from a Late Bronze Age settlement bordering the Lake of Bourget (Savoie, France) dated to *c*. 1100–900 B.C. and from the Iron Age Iberian settlement of Siriguarch near Alcañiz (Aragón, Spain) from *c*. 700–600 B.C. [70,110]. The traces of grain weevils at Arene Candide, earlier than those of Bagnoles, suggest that the spread of this pest began as early as the Neolithic through long-distance grain exchanges with the eastern Mediterranean. This is supported by the early finds of grain weevils in Greece at the sites of Dispilio (*c*. 5780–5720 B.C.) and Servia (*c*. 4500–4200 B.C.) [62,111]. A great number of grain weevils and other pest remains appear when the conditions of conservation are favourable in prehistoric storage structures. Various grain stores of this type have been explored. An example is the Prehispanic (*c*. 600–1450 A.D.) granary of La Fortaleza (Gran Canaria), which is carved into volcanic rock [112]. In addition to containing grain and legumes, this granary yielded the remains of 1637 grain weevils and other primary and secondary pests. Grains, legumes, and dried fruits were probably stored in these artificial caves over long periods of time, perhaps even years. Furthermore, the numerous remains of grain weevils identified at the Iberian Iron Age settlement of Siriguarch (Aragón) from *c*. 700–600 B.C. also come from a grain store. Noteworthy among the finds is a complete beetle in a grain seed [110]. In any case, grain storage features are hardly preserved at Les Bagnoles. It is possible, however, that certain larger sunken features referred to as silo-pits may have served to store grain [23,76].

While the number of pests in the grain stores of the two archaeological sites cited above can be indicative of high pest pressure, it is difficult to extrapolate the actual level of contamination of the three wells at Les Bagnoles based only on a few finds of grain weevils. These pests are very small and incapable of flight, suggesting a passive yet unsystematic introduction into the wells, a notion confirmed by their random distribution. Therefore, the beetles at Les Bagnoles probably found their way into the wells by means of rotten grains or, more rarely, in the straw of dungheaps [102]. Lastly, it is not possible to rule out that the grain weevils entered the well with human excrement. Osborne, in a very clear self-experiment, was able to prove in 1983 that when grain weevils are consumed by humans, their remains can be found in human excrement [113]. It therefore cannot be excluded that its presence resulted from the consumption of contaminated cereals by the inhabitants of Les Bagnoles. On the other hand, the great number of cereal chaff remains in the dung fragments rather suggest their link to ruminants.

The highest number of grain weevils in well 990 contrasts with that of later well 994. It is possible that this decrease is connected to an increase in the cultivation of glume wheat (einkorn) from this phase onwards [12]. Climatic change and cultural contacts are among the arguments serving to explain the increase of cultivation of glume wheat. A reaction to grain weevil infestation could likewise be behind this change. Perhaps attempts to limit grain weevil infestation was carried out by switching to glume wheat, as its grains, contrary to naked wheat, remain in the spikelet (where glumes encase the grain) during storage, rendering it much more difficult for the grain weevil female to drill a hole through the coats of their seeds with their mouthparts [63,114,115]. This change towards glume wheat would nonetheless lead to increasing the capacity of underground silos, as storage in the spikelet requires greater volume. However, calculations of the dimensions of storage pits in southern France in the late Middle Neolithic reveal a slight decrease of volume [12,116,117]. Several notions have been advanced concerning this issue, such as more diverse storage options [12]. Downsizing grain stores could have indeed been an additional measure against storage pests. Small, decentralised silos, contrary to large, central features serving several households, may have prevented the spread of pests within a settlement and protected parts of the stocks [103]. Nevertheless, the applicability of these considerations to Les Bagnoles is currently hard to test due to the fact, as already mentioned, of the scant number of grain silos.

Despite the scarce evidence of grain weevils, it is clear that the Neolithic communities could not permit their grain stocks to be infested by this pest, as it would not only render it unsuitable for consumption but delete the reserves intended for future sowing. Moreover, it is rather unlikely that infested or contaminated grain was regularly fed to the cattle, as they develop considerable rumen problems over time because their sensitive stomachs cannot tolerate the chitin of the insects’ exoskeletons over the long run and because grain contaminated by grain weevils is often also infested with mould [63,103]. However, it must be emphasised once again that grain weevil remains from the three wells of Les Bagnoles do not necessarily reflect the actual frequency of the pest or the extent of its distribution within Neolithic settlements.

Apart from grain weevils, well 990 also contained the remains of either a pea weevil or broad bean weevil (unclear identification). However, it is precisely this well that yielded a relatively large number of legume remains [12], for the most part peas (*Pisum sativum*) and one broad bean (*Vicia faba*) seed. The presence of pea weevils or broad bean weevils is therefore not surprising. In spring, the females of each of these species can fly and lay their eggs on immature pods in the fields. After the larvae hatch, they drill into the pod and migrate to the nuclei, where they develop [102]. Young beetles hatch in autumn and fly once again to the fields. Hence, these remains do not necessarily have to linger in the settlements and ultimately appear among its waste. However, it is impossible to estimate whether the seed beetle population in the legume fields of Les Bagnoles was large enough to provoke real damage to the crops of peas or broad beans.

It is also difficult to assess whether the low occurrence of the ground beetle (*Zabrus tenebrioides*) in well 994 is sufficient to classify it as a pest. These widespread and naturally occurring beetles probably attained the settlement in the stalks of grain. Their imago’s, in fact, do not cause any particular damage to winter grain. It is their larvae that are the actual pests, as they gnaw the young leaves and shoots of the cereal plants [104]. Because the larvae live in earthen passages in the fields and do not attain the settlement, it is not possible to assess their frequency in the fields and the damage they provoke.

The current study allows the strong assumption that the settlement of Les Bagnoles suffered infestation from different pest species. Yet, how early farmers took precautions to prevent, monitor, and control pests (pest management) unfortunately remains uncertain or is only the object of speculation. In general, small-scale agriculture with a diverse range of crops represent good courses of action to counter massive pest infestation [118,119]. Of course, this does not prevent the different pests from selectively consuming plants either in the field or in storage. The entire food base of the Neolithic population, however, was not completely endangered when adopting this type of plant cultivation. The loss of part of the grain or legume crop stocks can be compensated by other crop stocks.

The means applied to the storage of grain and legumes can also have an impact on infestation. It is likewise conceivable that certain physical barriers were created to keep rodents away from either the supplies or the fields [56]. That this was not always successful can be gleaned from the wood mouse finds in the granary of the Neolithic lakeshore settlement Chalain 3 in eastern France [48]. It is also conceivable that dogs were trained to ward off rodents, an assumption advanced for the Neolithic settlement of Orkney [42,43]. The use of herbal repellents [34], for example, dwarf elder (*Sambucus ebulus*), which was found in all wells but in higher numbers in wells 250 and 990 [24,25], is not proven so far. However, according to literature, there is evidence that it serves against infestation by rodents [120,121]. Setting traps is also entirely conceivable, albeit not yet proven. A special precaution against primary storage pests such as the grain weevil is storing unthreshed grain in their spikelets. However, this also implies cultivation of hulled cereals (and not only naked wheat/barley). Another very effective mechanical protection against insect pests is sprinkling the grains with dust or ash. The result is that the minerals contained in the dust and ash enlarge the body surface of the insects, leading them to dry out and die due to a loss of water [115,122]. Another useful side effect of the sprinkling seeds with ash or dust is the reduction of fungal attacks [122]. There are also a number of herbal repellents that, at times, drive away or harm pests. These include wormwood (*Artemisia absinthium* L.), dill (*Anethum graveolens* L.), marjoram (*Origanum vulgare* L.), rosemary (*Salvia rosmarinus* Spenn.), and laurel (*Laurus nobilis* L.) [123,124,125]. While the use of dill in well 990 and marjoram in wells 990 and 994 [24,25] as repellents is speculative, there is archaeological evidence of the use of these repellents, notably laurel in the grain stores of La Fortaleza of Gran Canaria (*c*. 600–1450 A.D.) [123].

## 5. Conclusions

The study of the remains of small mammals and invertebrates of Les Bagnoles definitely confirms the grain weevil as a storage pest. The study likewise highlights that potential pests such as wood mice, seed beetle, and corn ground beetle were also present at the Middle Neolithic settlement. The grain weevil formed part of the fill of all three wells and hence can be dated to both Middle Neolithic phases. Wood mice presumably appear from the second Middle Neolithic phase as potential storage pests. The same can be said of both pre-harvest seed and corn ground beetles. The crop shift observed at Les Bagnoles after 4000 cal B.C. (and in other regions of the western Mediterranean) may well have to do with a response to the (long-lasting) problem of crop pests. It is conceivable that this change played a role in reducing pest populations and thus improving food security for Neolithic groups. It is therefore likely that pests were more common in Neolithic settlements than what is suggested by the archaeozoological evidence. Thus, every further entomological investigation serves to shed light on the distribution of the different invertebrate pests. In addition, further indications of pest infestation can be collected when analysing plant remains bearing animal gnaw marks and insect boreholes, especially in the cases where there are no animal remains. Furthermore, finds of indigenous rodents not only point to pest infestation but can also help gain a better grasp of the distribution of other invasive species (e.g., wood mouse vs. house mouse). The same applies to the spread of different insect species. The results of the current study in any case demonstrate that delving into the theme of risk management of early farming communities requires exploring the role of pests. In fact, pests in the Neolithic represented a form of threat to food security alongside other factors, such as climate, weather, soil conditions, erosion, and limited water resources. It is possible that adopting other strategies besides changes in crop cultivation and intensification in crop cultivation (e.g., through fertilisation), biodiversity (diversification) in the fields, or varying the volume of storage (e.g., smaller rather than large central stores) also aimed at reducing pest infestation. Investigation into invertebrates and rodents can thus offer a more integral portrait of crop cultivation, its associated risks, and potential adaptation strategies. Unfortunately, poor preservation and lack of a research tradition impede tracing if similar pests existed at other sites in southern France or elsewhere in the western Mediterranean and their frequency in subsequent centuries. These are topics of future research that are essential if the appropriate sites and contexts, notably waterlogged deposits, are discovered.

## Figures and Tables

**Table 1:** Radiocarbon dates of wells 250, 990, and 994 of Les Bagnoles. ETH-113016 * combines two dates from the same sample.

Structure	Sample	Specie	Lab Code	BP	±	δ^13^C (‰)	C/N	Method	cal B.C. 2σ	Observations	Reference
250	Grain	Cerealia	ETH-60867	5331	27	−21.4	nd	AMS	4314−4051		Martínez-Grau et al., 2020 [73]
250	Fruit	*Corylus avellana*	ETH-60868	5306	27	−24	nd	AMS	4241−4047		Martínez-Grau et al., 2020 [73]
250	Grain	Cerealia	ETH-60869	5334	27	−20.5	nd	AMS	4316−4052		Martínez-Grau et al., 2020 [73]
250	Grain	*Triticum* sp.	ETH-60870	5302	31	−22.4	nd	AMS	4242−4001		Martínez-Grau et al., 2020 [73]
250	Grain	Cerealia	ETH-60871	5323	31	−24.2	nd	AMS	4312−4049		Martínez-Grau et al., 2020 [73]
250	Grain	*Triticum* sp.	POZ-64775	5400	40	nd	nd	AMS	4344−4061		Martínez-Grau et al., 2020 [73]
990	Grain	*Triticum aestivum/durum/turgidum*	ETH-88901	5226	25	−24.6	18.62	AMS	4216−3969		Martínez-Grau et al., 2020 [73]
990	Grain	*Triticum aestivum/durum/turgidum*	ETH-88904	5213	25	−24.8	18.96	AMS	4157−3965		Martínez-Grau et al., 2020 [73]
990	Bone	*Apodemus*/*Muridae*	ETH-113018	5161	33	−19.8	2.8	GIS	4046−3812		this paper
994	Grain	*Triticum monococcum*	ETH-88902	5027	26	−16.3	36.72	AMS	3947−3712		Martínez-Grau et al., 2020 [73]
994	Grain	*Triticum aestivum/durum/turgidum*	ETH-88903	5874	25	−23.7	40.75	AMS	4828−4689	outlier	Martínez-Grau et al., 2020 [73]
994	Grain	*Triticum monococcum*	ETH-96173	5096	28	−23.7	15.3	AMS	3967−3799		Jesus et al., 2021 [12]
994	Bone	*Apodemus*	ETH-113016 *	5672	52	−	−	GIS	4673−4364	outlier	this paper
994	Bone	*Apodemus*/*Muridae*	ETH-113017	5480	92	−23.2	0	GIS	4531−4052	outlier	this paper

**Table 2:** Number of identified specimens (NISP) and minimal number of individuals (MNI) of small mammals from wells 250, 990, and 994.

	Structure	Well 250			Well 990			Well 994			Total		
	Number of Samples	5			9			4			18		
	Sample Volume	78 L			346.8 L			163.5 L			588.3 L		
		NISP	NISP	MNI	NISP	NISP	MNI	NISP	NISP	MNI	NISP	NISP	MNI
		*n*	%		*n*	%		*n*	%		*n*	%	
Small mammals indet.		26			816			77			919		
**Carnivora**													
Least weasel	*Mustela nivalis*				2	0.2	1				2	0.2	1
Least weasel/Stoat	*Mustela niv.*/*ereminea*				58	7	1				58	5.6	1
**Rodentia**													
Murid/Vole	Muridae/Arvicolinae	14	54		401	48.2		51	40		466	45.1	
Woodmouse	*Apodemus* cf. *sylvaticus*	3	12	1	340	40.9	42	67	53	4	410	39.7	47
Vole	Arvicolinae	2	2		6	0.7		5	4		13	1.3	
Fieldmice	*Microtus* sp.				9	1.1		1	1		10	1.4	
Mediterranean pine vole	*Microtus duodecimcostatus*				6	0.7	2				6	0.6	2
Field vole	*Microtus agrestis*				2	0.2	1				2	0.2	1
**Eulipotyphla**													
Shrews	Soricidae	3	12		4	0.5					7	0.7	
Red-toothed shrews	Soricinae	1	4								1	0.1	
White-toothed shrews	Crocidurinae	2	8		1	0.1		1	1		4	0.4	
Greater white-toothed shrew	*Crocidura russula*				1	0.1	1	1	1	1	2	0.2	1
Mole	*Talpa* spec.	1	4		2	0.2	1				3	0.3	1
total identified		26	100		832	100		126	100		984	100	
*n* remains/liter sample vol.		0.7			4.7			1.2			1.7		

**Table 3:** Invertebrate pest species and invertebrate groups from wells 250, 990, and 994. Number of identified specimens (NISP) and extrapolated NISP (high) depending on the sample size.

	Structure	Well 250		Well 990		Well 994	
	Number of Samples	11		9		6	
	Sample Volume	148.5		57.8		84.2	
		NISP	high	NISP	high	NISP	high
**Pest insects**							
Carabidae/ground beetles	*Zabrus tenebrioides* (Goeze 1777)					4	4
Chrysomelidae/leaf beetles	*Bruchus* sp. (Linnaeus 1767)			1	1		
Curculionidae/weevils	*Sitophilus granarius* (Linnaeus 1758)	8	30	29	42	15	21
**Insecta**							
Lepidoptera/butterflies/moths		1	2			2	6
Hymnoptera/sawflies/waspe/bees/ants		25	197	21	70	186	427
Coleoptera/beetles		1080	4442	1880	3442	3792	5885
Trichoptera/caddisflies		6	29				
Diptera/true flies/mosquitoes		30	149	46	70	6	11
**Archanida**							
Araneae/spiders		3	32	1	1	8	13
Acari/mites		1	1				
Oribatida/moss mites		3	20	6	15	23	64
**Invertebrates indet**		358	2796	402	527	2841	5095
**total**		1515	7698	2386	4168	6877	11,526

## Data Availability

The studied microfaunal remains are accessible at the Integrative Prehistory and Archaeological Science (IPAS), Basel University, Basel, Switzerland. The datasets generated, analysed, and used in this study will be provided by the two corresponding authors on request.

## Supplementary Materials

The following supporting information can be downloaded at: https://www.mdpi.com/article/10.3390/ani12121511/s1, Figure S1: Section of well 250 (a), 990 (b), and 994 (c) with height information (m) and sequence of samples from top to bottom, dug spits (20–10 cm artificial levels). The investigation of small mammals and invertebrates was carried out in the lowest, waterlogged layers. US = stratigraphic unit. From: van Willigen et al., 2020a, simplified; Table S1: Summery table of semi quantified elements present in the organic fractions of the sieved sediment samples with an interpretation of the quality of preservation (see criteria in [79], total volume (L) of sediment samples and the examined sample volumes and fractions for invertebrate and small mammal remains); Figure S2: Single plots of the radiocarbon dates of 250, 990, and 994 wells. In red are indicated the *Apodemus* and *Apodemus*/*Muridae*. Calibration with OxCal v4.4.4 [80] and IntCal20 atmospheric curve [81]; Figure S3: Contemporary test results for 250, 990, and 994 wells.
