# Supplementary material for: Small Animals, Big Impact? Early Farmers and Pre- and Post-Harvest Pests from the Middle Neolithic Site of Les Bagnoles in the South-East of France (L’Isle-sur-la-Sorgue, Vaucluse, Provence-Alpes-Côte-d’Azur)
Source: Animals (Basel). 2022 Jun 9;12(12):1511. doi: 10.3390/ani12121511 (PMC9219518; doi:10.3390/ani12121511)
Supplement: Supplementary file 1 [file animals-12-01511-s001.zip › animals-1669765-supplementary.pdf]

Supplementary Materials:

Figure S1 a, b, c: Section of well 250 (a), 990 (b) and 994 (c) with height information (m) and sequence of samples from top to bottom, dug spits (20-10 cm artificial levels). The investigation of small mammals and invertebrates was carried out in the lowest, waterlogged layers. US = stratigraphic unit. From: van Willigen et al. 2020 [23] simplified.

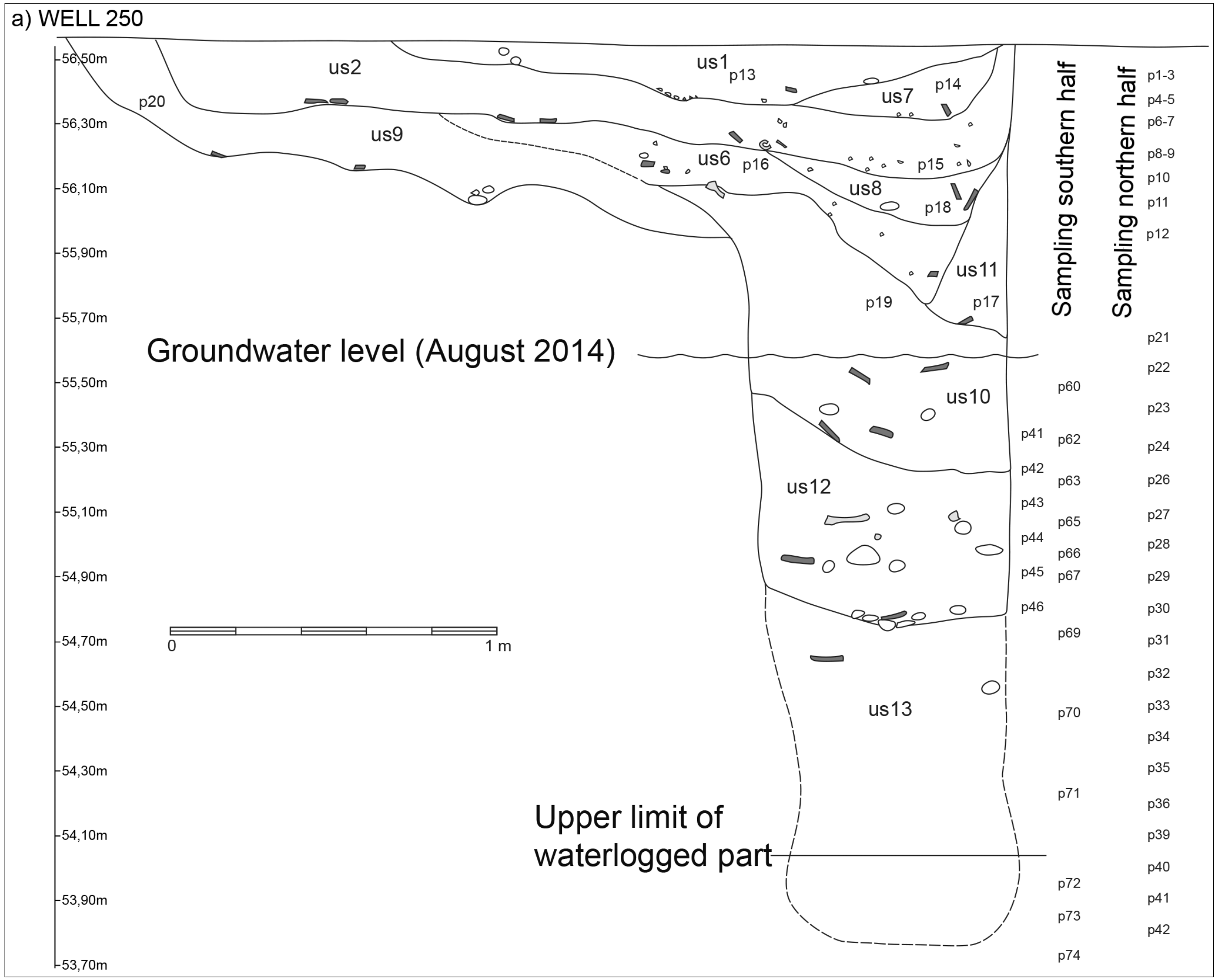

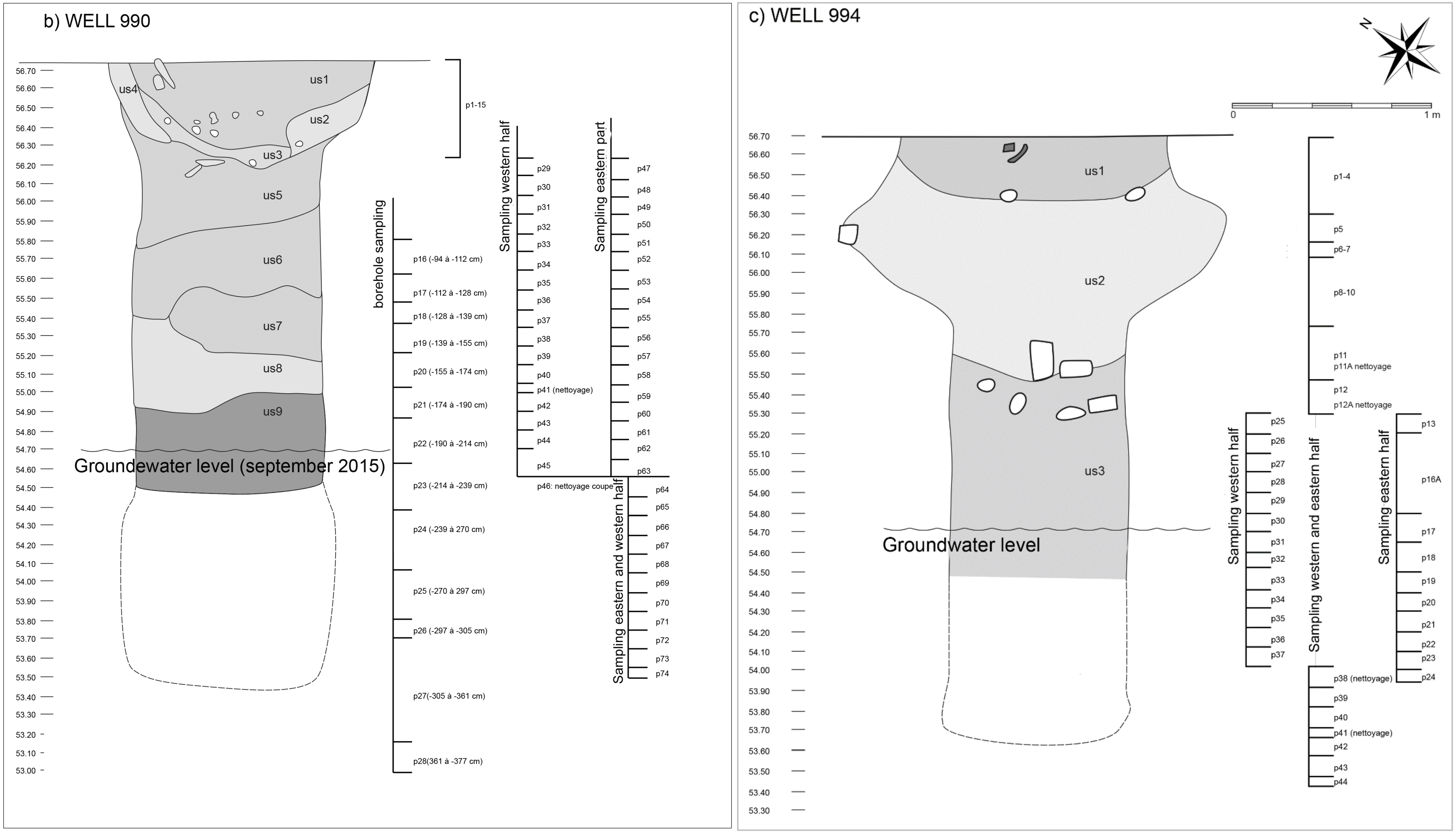

b) WELL 990
56.70
56.60
56.50
56.40
56.30
56.20
56.10
56.00
55.90
55.80
55.70
55.60
55.50
55.40
55.30
55.20
55.10
55.00
54.90
54.80
54.70
54.60
54.50
54.40
54.30
54.20
54.10
54.00
53.90
53.80
53.70
53.60
53.50
53.40
53.30
53.20
53.10
53.00
us1
us2
us3
us4
us5
us6
us7
us8
us9
Groundewater level (september 2015)
p1-15
borehole sampling
p16 (-94 à -112 cm)
p17 (-112 à -128 cm)
p18 (-128 à -139 cm)
p19 (-139 à -155 cm)
p20 (-155 à -174 cm)
p21 (-174 à -190 cm)
p22 (-190 à -214 cm)
p23 (-214 à -239 cm)
p24 (-239 à 270 cm)
p25 (-270 à 297 cm)
p26 (-297 à -305 cm)
p27(-305 à -361 cm)
p28(361 à -377 cm)
Sampling western half
p29
p30
p31
p32
p33
p34
p35
p36
p37
p38
p39
p40
p41 (nettoyage)
p42
p43
p44
p45
p46: nettoyage coupe
Sampling eastern part
p47
p48
p49
p50
p51
p52
p53
p54
p55
p56
p57
p58
p59
p60
p61
p62
p63
Sampling eastern and western half
p64
p65
p66
p67
p68
p69
p70
p71
p72
p73
p74
c) WELL 994
N
0
1 m
56.70
56.60
56.50
56.40
56.30
56.20
56.10
56.00
55.90
55.80
55.70
55.60
55.50
55.40
55.30
55.20
55.10
55.00
54.90
54.80
54.70
54.60
54.50
54.40
54.30
54.20
54.10
54.00
53.90
53.80
53.70
53.60
53.50
53.40
53.30
us1
us2
us3
Groundwater level
p1-4
p5
p6-7
p8-10
p11
p11A nettoyage
p12
p12A nettoyage
Sampling western half
p25
p26
p27
p28
p29
p30
p31
p32
p33
p34
p35
p36
p37
Sampling western and eastern half
p38 (nettoyage)
p39
p40
p41 (nettoyage)
p42
p43
p44
Sampling eastern half
p13
p16A
p17
p18
p19
p20
p21
p22
p23
p24

Table S1: Summery table of semi quantified elements present in the organic fractions of the sieved sediment samples with an interpretation of the quality of preservation (see criteria in [79], total volume (l) of sediment samples and the examined sample volumes and fractions for invertebrate and small mammal remains.

							Observations during sorting organic fractions						Invertebrate remains		Small mammal remains	
Well	Sample Nr.	Maximum depth (m asl)	Minimum depth (m asl)	Sediment sample total volume (l)	Pretreatment	Water-logged preser-vation	Dung	Quality preser-vation (plant remains)	Molluscs	Bones	Insects	Biotur-bation (roots)	Examined volume (l) organic fraction	Analysed organic fractions for	Examined volume (l) inorganic fraction	Analysed inorganic fractions
250	24	55.47	55.37	17	Freezing	yes		very bad					17	0.35 mm		
250	27	55.27	55.17	14	-	yes		very bad	abundant	abundant	present		14	0.35 mm		
250	28	55.17	55.07	12.5	-	yes		very bad	abundant	abundant			12.5	0.35 mm		
250	32	54.77	54.67	28	Freezing+Calgon	yes	charred	very bad					28	0.35 mm		
250	33	54.67	54.57	26.5	Freezing+Calgon	yes		very bad	abundant	present	present		26.5	1/0.35 mm		
250	34	54.57	54.47	29	Freezing+Calgon	yes		very bad	abundant	abundant	present		29	1/0.35 mm		
250	36	54.37	54.27	7.5	Freezing	yes	present	medium			present	yes	7.5	1/0.35 mm		
250	37	no info	no info	3	Freezing	yes		low				yes	3	0.35 mm		
250	38	54.27	54.17	26	Freezing+Calgon	yes	present	medium			present	yes	6	1/0.35 mm		
250	39	54.17	54.07	29.5	Freezing	yes	present	medium	present	medium	present	yes	6	4/1/0.35 mm		
250	40	54.07	53.97	21.5	Freezing	yes	present	good	abundant		present	yes	6	4/1/0.35 mm	15.5	4/1 mm
250	41	53.97	53.87	23.5	Freezing	yes	present	good	present		present	yes	7	1/0.35 mm	23	4/1 mm
250	42	53.87	53.8	14	Freezing	yes	present	medium	present		present	yes	6	1/0.35 mm	8	4/1 mm
250	73	53.97	53.77	23.5	Freezing	yes	present	medium		present		yes			23.5	4/1 mm
250	74	53.77	no info	8	Freezing	yes	present	medium	abundant	present	abundant	yes			8	4/1 mm
990	25	53.8	54.06	0.9	Calgon	yes	abundant	medium	present	abundant	abundant	yes			0.9	4/1 mm
990	26	53.72	53.8	0.4	Calgon	yes				present	abundant		0.4	4/1/0.35 mm	0.4	4/1 mm
990	28	53	53.16	0.4	Calgon	yes				present	present		0.4	4/1/0.35 mm		
990	68	54.06	54.16	59	-	no					present	yes	8	0.35 mm	50	4/1 mm
990	69	53.94	54.06	28	-	yes	abundant	good			abundant	yes	7	1/0.35 mm	28	4/1 mm
990	70	53.84	53.94	88	Calgon	yes	abundant	good		present	very abundant	yes	9	4/1/0.35 mm	88	4/1 mm
990	71	53.76	53.84	84.5	-	yes	abundant	good		present	abundant		7	4/1/0.35 mm	63.5	4/1 mm
990	72	53.66	53.76	78.5	-	yes	present	good			present	yes	8	4/1/0.35 mm	56	4/1 mm
990	73	53.58	53. 66	26	Calgon	yes	present	good	present	abundant	abundant		9	4 mm	26	4/1 mm
990	74	53.5	53.58	34	-	yes	very abundant	excellent			very abundant		9	4/1/0.35 mm	34	4/1 mm
994	20	54.3	54.42	0.2	Freezing	yes	no	low	no	no	abundant		0.2	1/0.35 mm		
994	41	53.7	53.72	31.5	Freezing +Calgon	yes	no	low	abundant	present	very abundant		16	1/0.35 mm	31.5	4/1 mm
994	42	53.6	53.7	53.5	Freezing+Calgon	yes	present	medium	abundant	abundant	abundant	yes	16	4/1/0.35 mm	53.5	4/1 mm
994	43	53.5	53.6	54.5	Freezing+Calgon	yes	no	low	abundant	present	abundant		17	4/1/0.35 mm	47	4/1 mm
994	44	53.65	53.5	32.5	Freezing	yes	no	low	abundant		abundant		18	4/1/0.35 mm	23.5	4/1 mm
994	45	53.6	no info	17	Freezing	yes	no	low	abundant		abundant		17	4/1/0.35 mm	8	4/1 mm

Figure S2: Single plots of the radiocarbon dates of 250, 990 and 994 wells. In red are indicated the *Apodemus* and *Apodemus*/*Muridae*. Calibration with OxCal v4.4.4 (Bronk Ramsey 2009) [80] and IntCal20 atmospheric curve (Reimer et al. 2020) [81].

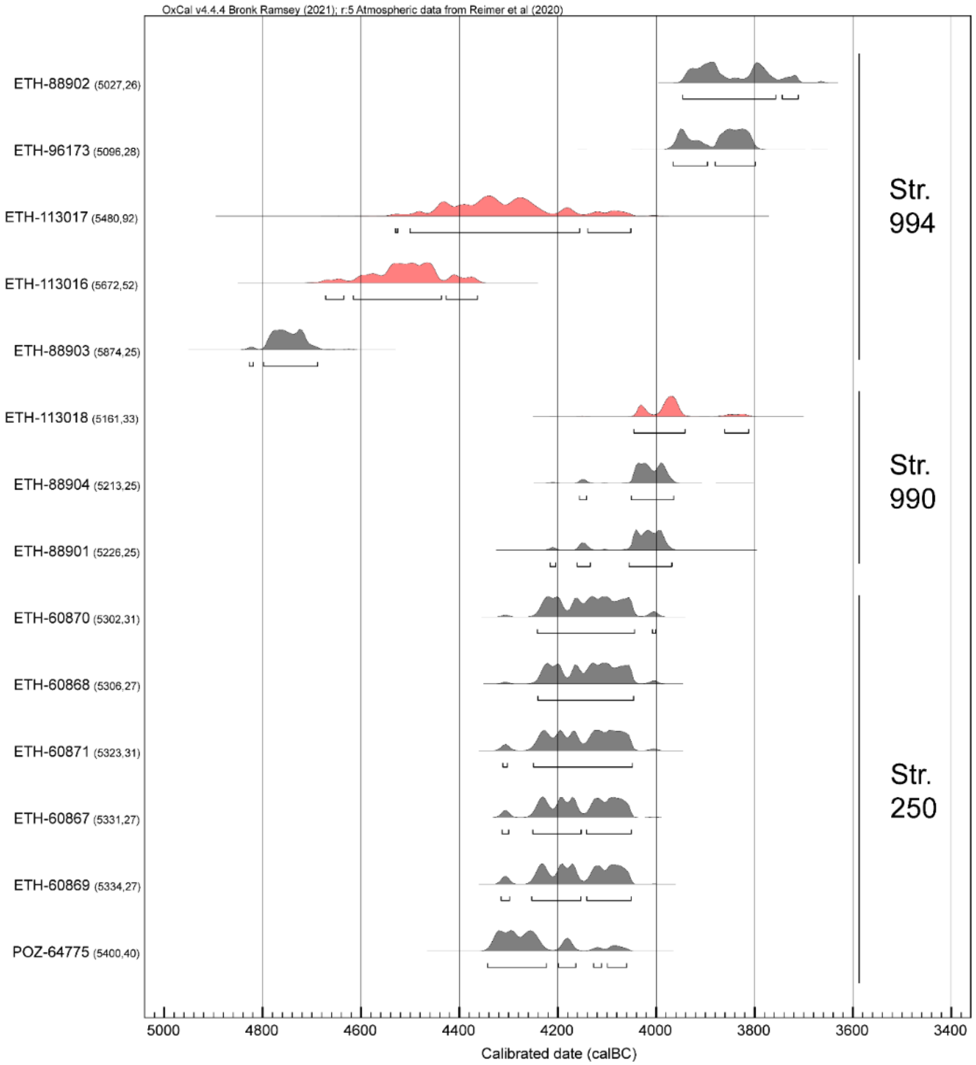

Figure S3: Contemporary test results for 250, 990 and 994 wells

well 250

Structure	Sample	Specie	Lab code	BP	±	$\delta^{13}C$ (‰)	C/N	Method	cal BC 2σ	Observations	Reference
250	Grain	Cerealia	ETH-60867	5331	27	-21.4	nd	AMS	4314 -4051		Martínez-Grau et al. 2020
250	Fruit	*Corylus avellana*	ETH-60868	5306	27	-24	nd	AMS	4241 -4047		Martínez-Grau et al. 2020
250	Grain	Cerealia	ETH-60869	5334	27	-20.5	nd	AMS	4316 -4052		Martínez-Grau et al. 2020
250	Grain	*Triticum* sp.	ETH-60870	5302	31	-22.4	nd	AMS	4242 -4001		Martínez-Grau et al. 2020
250	Grain	Cerealia	ETH-60871	5323	31	-24.2	nd	AMS	4312 -4049		Martínez-Grau et al. 2020
250	Grain	*Triticum* sp.	POZ-64775	5400	40	nd	nd	AMS	4344 -4061		Martínez-Grau et al. 2020

X^2 test	df	T	p-value (5%)		BP	SD	cal BC (95.4%)
well 250	2	4.7	11.1		5327	13	4236-4057

X2 test

df = 5 T = 4.7 p-value (5%) = 11.1

well 250 = 5327BP, 13SD = 4236-4057 cal BC (95.4%)

well 990

Structure	Sample	Specie	Lab code	BP	±	$\delta^{13}C$ (‰)	C/N	Method	cal BC 2σ	Observations	Reference
990	Grain	*Triticum aestivum /durum /turgidum*	ETH-88901	5226	25	-24.6	18.62	AMS	4216 -3969		Martínez-Grau et al. 2020
990	Grain	*Triticum aestivum /durum /turgidum*	ETH-88904	5213	25	-24.8	18.96	AMS	4157 -3965		Martínez-Grau et al. 2020
990	Bone	*Apodemus* / *Muridae*	ETH-113018	5161	33	-19.8	2.8	GIS	4046 -3812	C/N ratio out of range	this paper

X^2 test	df	T	p-value (5%)		BP	SD	cal BC (95.4%)
well 990	2	2.6	6.0		5207	16	4046-3972

X2 test

df = 2 T = 2.6 p-value (5%) = 6.0

well 990 = 5207BP 16SD = 4046-3972 cal BC (95.4%)

well 994

Structure	Sample	Specie	Lab code	BP	±	$\delta^{13}C$ (‰)	C/N	Method	cal BC 2σ	Observations	Reference
994	Grain	*Triticum monococcum*	ETH-88902	5027	26	-16.3	36.72	AMS	3947 -3712		Martínez-Grau et al. 2020
994	Grain	*Triticum monococcum*	ETH-96173	5096	28	-23.7	15.3	AMS	3967 -3799		Jesus et al. 2021

X^2 test	df	T	p-value (5%)		BP	SD	cal BC (95.4%)
well 994	1	3.3	3.8		5059	20	3949-3796
